# What is known about the patient's experience of medical tourism? A scoping review

**DOI:** 10.1186/1472-6963-10-266

**Published:** 2010-09-08

**Authors:** Valorie A Crooks, Paul Kingsbury, Jeremy Snyder, Rory Johnston

**Affiliations:** 1 Department of Geography, Simon Fraser University, 8888 University Drive, Burnaby, British Columbia, Canada; 2 Faculty of Health Sciences, Simon Fraser University, 8888 University Drive, Burnaby, British Columbia, Canada

## Abstract

**Background:**

Medical tourism is understood as travel abroad with the intention of obtaining non-emergency medical services. This practice is the subject of increasing interest, but little is known about its scope.

**Methods:**

A comprehensive scoping review of published academic articles, media sources, and grey literature reports was performed to answer the question: what is known about the patient's experience of medical tourism? The review was accomplished in three steps: (1) identifying the question and relevant literature; (2) selecting the literature; (3) charting, collating, and summarizing the information. Overall themes were identified from this process.

**Results:**

291 sources were identified for review from the databases searched, the majority of which were media pieces (*n *= 176). A further 57 sources were included for review after hand searching reference lists. Of the 348 sources that were gathered, 216 were ultimately included in this scoping review. Only a small minority of sources reported on empirical studies that involved the collection of primary data (*n *= 5). The four themes identified via the review were: (1) decision-making (e.g., push and pull factors that operate to shape patients' decisions); (2) motivations (e.g., procedure-, cost-, and travel-based factors motivating patients to seek care abroad); (3) risks (e.g., health and travel risks); and (4) first-hand accounts (e.g., patients' experiential accounts of having gone abroad for medical care). These themes represent the most discussed issues about the patient's experience of medical tourism in the English-language academic, media, and grey literatures.

**Conclusions:**

This review demonstrates the need for additional research on numerous issues, including: (1) understanding how multiple information sources are consulted and evaluated by patients before deciding upon medical tourism; (2) examining how patients understand the risks of care abroad; (3) gathering patients' prospective and retrospective accounts; and (4) the push and pull factors, as well as the motives of patients to participate in medical tourism. The findings from this scoping review and the knowledge gaps it uncovered also demonstrate that there is great potential for new contributions to our understanding of the patient's experience of medical tourism.

## Background

Medical tourism is becoming an increasingly popular option for patients looking to access procedures (typically via out-of-pocket payment) that are seemingly unavailable to them in their home countries due to lack of affordability, lack of availability, and/or lengthy waiting lists, among other reasons [1,2]. In its broadest conceptualization, medical tourism refers to "travel with the express purpose of obtaining health services abroad" (p.193) [3]. People wishing to access procedures such as cardiac, orthopaedic, dental, and plastic surgeries are going to key destination countries known to provide care for international patients [4]. For example, countries such as India, Singapore, and Thailand have become global leaders in the industry, providing services for patients from around the world. Brochures, websites, and other marketing materials promote the services of hospitals in these countries wanting to attract international patients [5]. Facilitators/brokers specializing in medical tourism further promote the practice, and offer services such as making travel bookings, assisting with selecting hospitals and surgeons abroad, and helping with completing paperwork to potential medical tourists [6].

The practice of medical tourism does not exist without criticism, particularly when involving patients from developed nations going to developing nations for procedures. It is thought to contribute to the commodification of health and health care by allowing those with the financial means to do so to purchase care that may be unavailable to other citizens [7]. The practice can also lead to international patients receiving a higher standard of care than residents of the country where it is being given [8]. Another criticism is that health service providers trained in countries with publicly-funded education systems who are involved in privately treating international patients are misdirecting the public funds that contributed to their training [9]. It has, however, been suggested that if the industry is properly regulated, medical tourism can provide a viable means by which developing countries can gain access to needed revenue and developed countries can lessen 'bottlenecks' in their health systems [10]. The presence of medical tourism hospitals in developing nations is also thought to lessen the international brain drain of health human resources by providing surgeons and others with access to advanced, high technology work environments [3].

Although estimates of the number of patients engaging in medical tourism each year vary widely, ranging from millions to tens of thousands, there has been speculation that growth in the industry will continue in the coming years [11-13]. Given the prominence of this global industry, research and media attention focused on it will also surely continue to grow. With projections of growth in the industry and the existence of significant criticisms about the practice, it is an opportune time to undertake knowledge syntheses to assess what exactly is known about medical tourism so as to ultimately inform research, government, and industry agendas alike. In the remainder of this article we take on this task, presenting the findings of a scoping review that addresses the question: what is known about the patient's experience of medical tourism? This article serves as the first attempt to draw together what is known about this issue, and thus is a valuable contribution to the burgeoning literature on medical tourism. In an attempt to be as comprehensive and inclusive as possible, multiple types of sources are included in the review, including: academic articles, newspaper and magazine articles, industry reports, and law reviews. Such inclusivity is central to the scoping review process in general, where the aim is to appreciate the breadth of knowledge that is available on a particular topic [14].

While there is no singular definition of medical tourism that has gained wide acceptance, in this article we place some widely acknowledged parameters on what it is understood to be in order to focus the scoping review. People who become ill or injured while traveling abroad and require hospital care are not thought to be medical tourists, nor are expatriates accessing care in the countries or regions in which they live. A survey run by the Thai government to assess the scope of its domestic medical tourism industry distinguished between international patients who were medical tourists, ill vacationers, and expatriates living in Thailand or a neighbouring country, which confirms the distinctions being used here [15]. Established cross-border care arrangements between countries are not forms of medical tourism. This is because out-of-pocket payments for the accessed care are not typically made under such arrangements, as is the case for medical tourists, and because these arrangements typically require referrals to be given for care that is not available locally based on collaborative arrangements between hospitals or care systems. Meanwhile, medical tourists can choose to go abroad for care without the referral of a physician. These distinctions are made elsewhere. For example, a World Health Organization report on cross-border care within Europe distinguishes between patients travelling *independently *(i.e., without referral) for care internationally, those who are sent abroad by their home systems in order to access specialized care that is not available locally, and those who live in border regions with traditions of sharing care across borders [16]. Further, the pursuit of complementary and alternative care abroad is not medical tourism; instead, it falls under the even broader rubric of health tourism. When taken together, these parameters result in achieving a focused understanding of medical tourism, whereby it occurs when patients *intentionally *leave their country of residence *outside of established cross-border care arrangements *in pursuit of non-emergency medical interventions (namely surgeries) abroad that are commonly paid for out-of-pocket. This typically includes staying abroad for at least part of the recovery period, whereby such post-discharge time can be spent at tourist resorts that cater to international patients [17-19].

## Methods

Broadly speaking, knowledge syntheses aim to collect and evaluate the current state of knowledge on a particular issue [14]. The scoping review is a knowledge synthesis technique that is most commonly used when: it is difficult to identify a narrow review question; studies in the reviewed sources are likely to have employed a range of data collection and analysis techniques; no prior synthesis has been undertaken on the topic; and a quality assessment of reviewed sources is not going to be conducted [14]. In this article the findings of a scoping review that meets all of these criteria is presented. The review poses the broad question: what is known about the patient's experience of medical tourism? The synthesis presented in this article follows the scoping review protocol set out by Arksey and O'Malley [14]. In the remainder of this section we outline the steps undertaken to complete the review.

### Identifying the Question and Relevant Literature

The first step was to develop the scoping question, which was done by holding a research team meeting to identify a potentially fruitful and also useful issue to focus on within the area of medical tourism. Next the team moved to delineate a search strategy that would lead to the identification of relevant literature. To do this, keywords were first identified based on review of relevant literature and ultimately team consensus. As depicted in Table 1, keywords probed five main categories: (1) focus; (2) what; (3) who; (4) why; and (5) where. Eight types of rationale were identified for the why category, as shown in Table 1. Known destination and departure countries were used to populate the where category.

**Table 1:** Scoping review keyword search strategy

Focus	What	Who	Why	Where
Medical tourism Health tourism	Surger* Elective surger* Surgical Procedure* Hospital* Clinic*	Patient Tourist	Decision making Factors Decision Attitudes Motivation Destination choice	Destination Brazil India Thailand South Africa Indonesia Cuba Mexico Philippines Singapore United States Canada
				
			Tour* Travel Vacation* Adventur*	
				
			Wait time Wait list Queue Speed	
				
			Value* Ethic*	
				
			Privat* Effects Two tier	
				
			Cost savings Affordability Savings Cost	
				
			Motivat* Perspective*	
				
			Distance Quality	

Following finalization of the keywords, a search strategy was created with the input of a librarian to scope the English-language academic, media, and grey literatures to achieve as much breadth as possible. Combinations of terms were rationally searched in 18 databases, summarized in Table 2, with a different search strategy being employed between academic and media databases. For academic databases, keywords across the five categories summarized in Table 1 were searched using Boolean operators in order to maximize the permutations of terms scoped. Certain combinations of keywords yielded unmanageably large and mostly irrelevant results. In these instances the search manager narrowed the results either by adding additional keywords or removing the term that had the broadest results to ultimately enhance the focus and relevance of the findings.

**Table 2:** Databases searched for scoping review

Database Type	Database	Temporal Period Covered
Academic	Academic Search Premier	1984 - 20/08/2009
	Ageline	1978 - 10/08/2009
	Biomed Central	no recorded start date - 10/08/2009
	Business Source Complete	no recorded start date - 20/08/2009
	Canada Research Index	1982 - 21/07/2009
	CINAHL	1982 - 20/07/2009
	CPI.Q	1988 - 22/10/2009
	EconLit	1969 - 9/08/2009
	Geobase	1980 - 9/08/2009
	Global Health	1973 - 10/08/2009
	Medline	1950 - 9/08/2009
	PAIS International	1972 - 9/08/2009
	PsycINFO	1887 - 10/08/2009
	Sociological Abstracts	1963 - 20/08/2009
	Web of Science	1900 - 10/08/2009
Media	Alternative Press Index	1991 - 20/07/2009
	CBCA Current Events	1982 - 21/07/2009
	Canadian Newstand	1985 - 22/10/2009
	Lexis Nexis	no recorded start date - 22/10/2009

As searching all media sources around the globe was not feasible, the team focused the media search on a particular country (Canada) to capture local, regional, and national coverage of medical tourism. Team had best access to media search databases for Canadian sources, which is why Canada was selected as the focus. It is, however, expected that the identified sources may replicate what some media coverage of the patient's experience of medical tourism is like in other prominent patient departure countries (e.g., the United States [US], the United Kingdom), keeping in mind that there are important differences in regulatory and health system environments operating between such countries that are likely to have influences on patients' experiences. Only the terms 'medical tourism' and 'health tourism' were searched in the media databases. The databases were searched broadly for these terms, and key North American sources known to frequently cover Canadian health services issues were also specifically searched within the Lexis Nexis database (namely the New York Times, Time inc., Globe and Mail, Associated Press, magazines, Washington Post, Toronto Star, Toronto Sun, CBC News). Sources of all types across the academic and media databases deemed relevant to the search were retrieved and organized using the Refworks bibliographic management program.

### Selecting the Literature

In order to select literature for inclusion in the review the team first searched titles and abstracts of the identified sources. All team members independently reviewed each title and abstract and consensus was sought as to whether or not to read sources in full, which were reviewed in batches given the large number of identified sources. '*Post hoc*' inclusion criteria were created and employed by the team at this step. The development of such criteria '*post hoc*' is central to the scoping review process as it is unlikely that researchers will be able to identify bases for exclusion at the outset, and this, in fact, is a key point of differentiation between the scoping and systematic review processes [14]. The team identified three bases for exclusion: (1) there was no focus on medical intervention, which included articles that dealt with health tourism more broadly such as international travel to healing spas; (2) there was an exclusive focus on 'reproductive tourism' or 'transplant tourism', as the medical intervention (if any) in such cases is not restricted to the international patient and thus raises separate considerations; and (3) there was an overly general focus on international trade in health services or cross-border care, where there seemed to be no explicit reference to medical tourism. Disagreements regarding whether or not a source should be included for full review were discussed among the team until consensus was reached. As the title and abstract review moved forward, the level of agreement among team members, which was already high from the outset, continued to increase.

Upon completion of the title and abstract review, included sources were reviewed in full. Titles and abstracts were not available for media sources and so they were not involved in the first round of inclusion/exclusion and were all reviewed in full. Further, included sources were hand searched and relevant sources not already gathered from the search databases were identified for full review. The three exclusion criteria created at the title and abstract review stage were applied at the full review stage, with one additional criterion being applied: if no 'informational points' (i.e., discrete pieces of information found within sources that contributed to answering the scoping question) were extracted from the source it was excluded. Two team members reviewed every source identified for full review. Sources were reviewed in batches, and upon completion of each new batch the team met to review decisions regarding the inclusion or exclusion of sources. As with the title and abstract review stage, any disagreement was resolved through seeking consensus among all members after discussion.

### Charting, Collating, and Summarizing the Information

To chart the informational points extracted from the sources, a spreadsheet was created and securely hosted online that was used by all team members. Details regarding publication information, study design (if relevant), and the sample (if relevant) were recorded, along with any informational points pertinent to the overall scoping question. These details were recorded independently by each reviewer for all of the sources, including those that were ultimately excluded. The extracted informational points were discussed during team meetings in order to work towards gaining an overall perspective on the themes emerging from the literature pertaining to the scoping question, which is essential to the charting process [14]. As discussed in the results section, four such themes were identified. Following this the extracted informational points in the spreadsheet were colour coded according to theme in order to assist with organizing the reporting of the scoping review findings. Finally, the team then worked together to identify important avenues for future research by identifying knowledge gaps.

## Results

**Figure 1:**
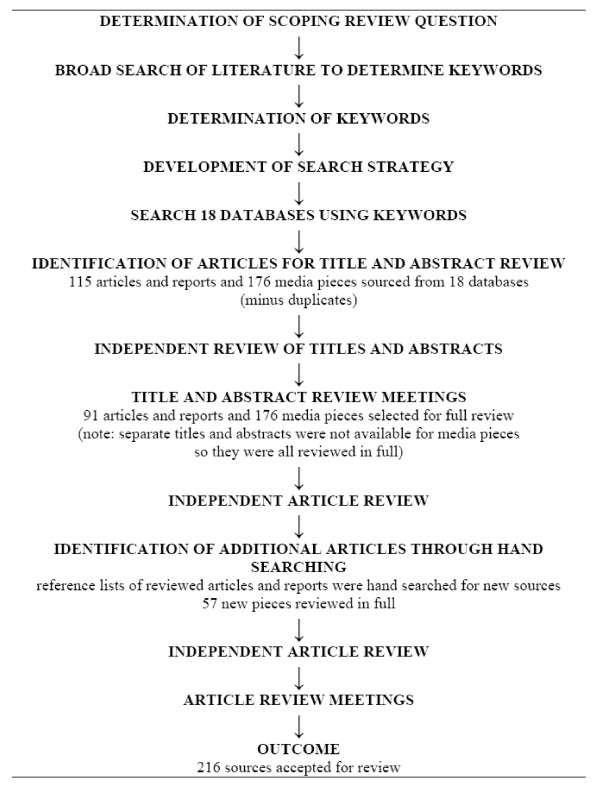
**Search strategy and results**.

Shown in Figure 1, 291 sources were identified for review from the databases searched, the majority of which were media pieces (*n *= 176). A further 57 sources were included for review after hand searching reference lists. Of the 348 sources that were reviewed either partially (title and abstract) or in full, 216 were ultimately included in the scoping review (a full list of included sources can be obtained by contacting the lead author). While this is a large number relative to some knowledge syntheses (e.g. many systematic reviews), as an aim of scoping reviews is to pose a broad question and then appreciate the extent of the literature on an issue from a variety of sources it is not unthinkable to include this many sources in a single review [14]. The included sources were authored in ten different countries (Australia, Canada, France, India, Malaysia, Norway, Spain, Thailand, US, and United Kingdom - many of which are prominent home or destination countries for medical tourists), and made explicit reference to a number of others (e.g., China, Cuba, Germany, Jordan, Mexico, Poland, Singapore, South Africa, Tunisia, United Arab Emirates, Yemen), which geographically reflects the state of interest in medical tourism.

Only a small minority of sources included in the scoping review were reporting on empirical studies that involved the collection of primary data (*n *= 5) [20-24]. Instead, they were mostly conceptual pieces, discussion papers, law reviews, commentaries, editorials, reviews, magazine and newspaper articles, reports, and business briefs published in a range of venues. The breadth of sources included in the review has allowed for perspectives from a number of sectors, including industry, health professionals, health service administrators, tourism operators, and academia, to be captured in the review. At the same time, most of the sources are heavily speculative or anecdotal in nature, relying on opinion instead of empirical facts. Because of the emerging nature of medical tourism, however, and the lag time involved in gathering and publishing empirical evidence, grey literature and media reports that offer preliminary glimpses were not excluded because they provide some of the only insights into the patient's perspective. Instead, caution was exercised in interpreting themes from the informational points extracted from the sources and a tentative tone is adopted below in sharing what was gleaned.

Though there was little dedicated focus on the patient's experience of medical tourism in the included sources, particularly that which had come from consulting directly with medical tourists themselves, when taken together they collectively contribute to answering the scoping review question. This contribution comes in the form of extracted informational points relating to the four themes identified across the sources, namely: (1) decision-making; (2) motivations; (3) risks; and (4) first-hand accounts. These four themes represent the most discussed issues about the patient's experience of medical tourism in the English-language academic, media, and grey literatures. In the remainder of this section we expand on these themes. Given the large number of sources included in the review, for manageability we cite no more than six at a time. While patients within the developing world do indeed travel abroad for health services, sometimes as medical tourists, the bulk of the sources focus on travel from developed to developing countries (referred to sometimes as north-south or west-east medical tourism) [20-22]. As such, in the remainder of the article we are referring to patients traveling from developed nations to developing ones unless otherwise stated, though certainly some of the findings are applicable to south-south patient flows.

### Decision-making

Three issues were most discussed with regard to decision-making: (1) push factors (i.e., things that drove patients away from care at home); (2) pull factors (i.e., things that drew patients to other countries); and (3) information sources consulted. The push factor noted most frequently was that of cost [25-30]. It was commonly reported that the high cost of out-of-pocket payments for procedures in patients' home countries likely pushes them to consider medical tourism [5,17,31-34]. Related to cost, a lack of insurance, or being underinsured, may also push people into medical tourism, in that if procedures cannot be covered by their insurance plans then they may be pushed abroad in search of more affordable care [5,17,35-38]. The other most frequently noted push factor was that of wait-times, with the promise of more timely care in other countries potentially drawing them abroad [17,39-43].

The pull factors identified in the reviewed sources were more numerous than the push factors. The most frequently discussed pull factor was quality. Patients can be pulled towards medical tourism hospitals that are renowned for the quality of service, care, and facilities they offer [1,20,26,32,40,44]. Language is also factored into decision-making, wherein patients are thought to be drawn to receiving care in places where hospital employees speak their language [1,45-48]. Related to this, the religious accessibility of medical tourism facilities and destination nations is another consideration that can pull patients to one location over another. More specifically, patients may seek out facilities that observe the same religious protocols they do [48,49]. The political climate of countries or regions may also pull people to receive care in particular locations, in that patients are unlikely to want to travel to places that are politically (or even culturally) unstable or inaccessible [20,50-52]. The vacation aspect of medical tourism serves as another pull factor, in that patients may be drawn to receive care in places they are interested in holidaying in [2,3,46,53-55].

Having access to information while decision-making about medical tourism is vital given the range of factors that patients needed to consider before committing to going abroad, such as the credentials of doctors [56-58]. The international marketing of facilities and procedures, including their costs, online, via facilitators/brokers, aids some patients in decision-making [13,27,59-62]. The presence of such marketing informs potential patients about treatment options, tourism opportunities, and other key pieces of information that assist with decision-making. The internet also offers patients other types of information about medical tourism. For example, websites created by former medical tourists to share their experiences can act as an information source for those at the decision-making stage [56]. Word-of-mouth is also an important information source, with some medical tourists having first learned about the potential for accessing procedures abroad from friends and family [6,20,34,63-65].

### Motivations

In addition to wanting to address a personal health need through surgery, the review captured three types of factors motivating patients' engagement in medical tourism: (1) procedure-based; (2) travel-based; and (3) cost-based. A procedure-based motivator noted in several sources is that patients may wish to pursue procedures abroad that are illegal or not available in their home countries [1,66-70]. For example, some patients can gain access to experimental procedures abroad that have yet to be approved for use by doctors in their local hospitals, such as stem cell therapies [71]. Related to this, medical tourism can enable access to specific expertise and specialization [44] as well as advanced technologies [22,66,72,73]. These factors may also be particularly motivating for middle- and upper-class residents of developing countries who can afford to pay for more sophisticated care abroad [25]. Furthermore, hearing success stories about positive outcomes from others, whether by word-of-mouth or online, can serve as a motivator for potential medical tourists [20].

As noted in the previous section, the potential for travel and tourism is something that patients may consider when decision-making about medical tourism. It is thus not surprising that certain travel-based factors may actually serve as motivations for ultimately deciding on medical tourism, including on the destination location. Such motivators are thought to be the increasing ease and affordability of international travel, the frequency of flights to major destinations, and the streamlining of visa procedures and expediting of applications for international patients [47,74-77]. The availability of facilitators/brokers to assist with making detailed arrangements, corresponding with doctors, and planning after care in certain departure countries (e.g., in Canada it is reported that there are at least 20 different facilitators/brokers) can serve as a motivator for those reluctant to have to make their own plans and bookings [6,29,36,72]. Another travel-based motivator may be the presence of package deals, in that their affordability and ease of booking again may appeal to those looking for guidance in planning [45,72,78].

Not surprisingly, cost and affordability were often discussed as potential motivations for patients' engagement in medical tourism. In countries that have public health care coverage, such as Canada, the (often inaccurate) perception among patients that they may receive a partial or full reimbursement for the travel and procedure costs incurred abroad is thought to motivate some to engage in medical tourism [40,73,79,80]. However, it was noted that restrictions were typically placed on the availability of reimbursements for planned procedures undertaken abroad via medical tourism and that out-of-pocket cost coverage is extremely rare [6,40,80-82]. A significant amount of the discussion coming out of the US, a country without a public health insurance plan, reveals that others' desires to keep patients' health care costs low may ultimately influence patients' choices regarding going abroad. More specifically, some employers and insurance companies are encouraging people to access surgeries abroad because costs, even when travel is factored in, are substantially lower than what would be incurred at home [1,64,83-85].

### Risks

Given the challenges that people may face when undergoing surgery or traveling abroad, it is not surprising that the risks of medical tourism, which combines both of these aspects, were discussed throughout the reviewed literature. Three categories of risk were covered: (1) risks to patients' health; (2) risks of travel; and (3) risks pre- and post-operatively in the home country. Risks that patients may be exposed to that can have clearly negative impacts on their health include: contracting an infection post-operatively while in the hospital, travelling during the recuperative period, and an inadequate blood supply being available on-site at the hospital to meet the patients' needs [35,86,87]. It is also thought that undertaking procedures that are illegal in patients' home countries or experimental may expose medical tourists to unknown health risks, which may be the very reason that these same surgical procedures are not being performed in their home countries [46]. Related to cost motivations and decision-making factors, there is also a broad concern that making clinical decisions based on procedure costs is risky and may have negative outcomes for medical tourists and their health [39].

While there are always risks associated with travel, two travel-related risks that are particularly relevant to medical tourists were highlighted in the literature. The first pertains to airline travel. More specifically, flying with a serious health condition either in advance of the surgery (i.e., while getting to the hospital) or post-operatively increases patients' susceptibility to deep vein thrombosis (or sometimes referred to as 'traveler's thrombosis' when occurring as a result of a long-haul flight) [20,36,88,89]. This occurs when blood clots form in deep veins and cause affected areas to swell. Patients not well enough to move around during the flight may have restricted blood flow to areas of the body, thus resulting in deep vein thrombosis [86]. In some instances the clots may dislodge and travel to the lungs, thereby causing a pulmonary embolism. It was also noted that being away from family, particularly during the recuperative period abroad, and the mental strain of travel may lead to the onset of psychological and/or emotional stress for medical tourists, thus posing as another type of travel-related health risk [5,13,21].

The risks of medical tourism are not restricted to occurring while patients are abroad, nor do they end upon check-out from the hospital. Patients may not seek advice from their regular doctors, or may go against their doctors' advice, regarding whether or not surgery is needed [19,71,90,91]. Related to this, patients' medical records may become discontinuous, in that there are not presently adequate systems in place for transferring health information between medical tourism hospitals and patients' home doctors [39,92,93]. This problem may be overcome by patients carrying their records with them overseas and bringing back new files from abroad for inclusion in their permanent records [65]. Some reviewed sources further suggested that there may also be health risks upon return due to a lack of after-care planning [42,89,94], or that after-care may be challenging due to informational discontinuity [85]. Another risk post-operatively that may be experienced upon returning home is that there is commonly little legal recourse for procedures for which complications have arisen [86,95,96]. This is primarily due to weak malpractice laws that exist in many destination countries [73,92,97]. A related risk is that some doctors in home countries may be reluctant to treat medical tourists upon returning home for fear that they will be sued for complications arising from procedures undertaken abroad in countries with limited options for legal redress [90].

### First-hand Accounts

First-hand accounts of medical tourists' experiences were found mostly in reports and media sources. These accounts typically focused on one of three things: (1) positive and negative aspects of medical tourism; (2) sensationalized issues; and (3) reports of post-recovery life. Topics often covered in these accounts ranged from reports of being satisfied with the care received [11] and the benefits and drawbacks of recovering in a relaxing tourist locale away from home [42,98] to the experience of deciding on a particular hospital [61]. The perspectives offered on these issues were quite broad, and covered both positive and negative experiences. On the more negative side, accounts covered details such as patients having to borrow heavily from family in order to afford to access care abroad [18] and concerns about being treated by foreign doctors who may speak a different language and have different care standards [2].

One of the more sensationalized topics shared in patients' published accounts of medical tourism were stories of their own and others' expectations of cleanliness and care quality in hospitals abroad. A patient reporting on care having been received in Thailand said: "...this is not a straw-village clinic with rusty scalpels!" (p. 388) [38]; meanwhile another who accessed dental surgery in China reported: "It was dubious when you looked at it [the clinic], but when you got into the place they were competent, intelligent, and did everything they had to do" (p. 68) [99]. It is not surprising that patients emphasized such issues in their accounts given that there were reports of having to counter others' perceptions of care abroad. As a woman from the US who had received surgery in Thailand explained: "They [friends, family, and others] roll their eyes up in their heads and say, 'I can imagine' and I say, 'no, you can't...I went down and had lunch at the Starbucks in the lobby of the hospital, came back up and the doctor had on his desk the most beautiful file, all bound with tabs and everything, with all the results of the tests that they had done'... Something like that, as you know, is impossible in America...I mean, it's inconceivable" (p. A6) [100]. Others shared this experience, including another US patient who said that: "when I told people I was having surgery in Southeast Asia, some looked at me like I was crazy. They were clearly imagining me in a straw hut with someone holding fishing line and tweezers" (p. P01) [101]. Others' accounts focused on how care at hospitals abroad was not as sterile as they had expected [102].

Retrospective, post-recovery reports were abundant in the media sources reviewed. In these accounts former medical tourists reflect, overwhelmingly positively, on their experience overall and the positive impact that receiving a procedure abroad has had for their health. A former Canadian medical tourist had this to say: "Life is too precious. I'm in my early 50s and I have lots of things to do in my life and one of them isn't lying at home in pain... I'm a Victoria firefighter. I have been for 29 years and I don't want to retire that way, you know, with a disability" (n.p.) [103]. Accessing surgery abroad enabled him to maintain his employment. Another Canadian retrospectively proclaimed "I think it's the best money I've ever spent" (n.p.) [104], with regard to having paid out-of-pocket for surgery in India.

## Discussion

Table 3 summarizes the themes and issues generated from the 216 sources included in this scoping review. The informational points extracted in the review, generated primarily from sources reliant on speculation, reveal how complex the patient experience is, in that it involves many components ranging from early decision-making about multiple factors to ensuring appropriate arrangements for post-operative care in one's home system. The informational points also suggest that multiple individuals are involved in shaping the patient experience, including: family and friends who may have shared their own successful experiences of receiving care abroad with potential medical tourists; surgeons, nurses and patient coordinators who have direct responsibility for how on-site care is delivered in destination hospitals; and medical tourism facilitators/brokers who work to assist patients at multiple points. In the remainder of this section we explore the implications of the key issues identified about the patient's experience of medical tourism, knowledge gaps that exist on this topic, and also the overall limitations of the scoping review process employed.

**Table 3:** Summary of extracted informational points

Themes Identified	Main Issues Covered in Reviewed Sources	Example
Decision-making	Push factors	High out-of-pocket costs for procedures in patients' home countries
	
	Pull Factors	Hospitals known to deliver high quality care
	
	Information Sources Consulted	Word-of-mouth

Motivations	Procedure-based motivations	Wanting access to procedures that are illegal or unavailable in home country
	
	Travel-based motivations	Ease of booking
	
	Cost-based motivations	Recommendation by employer or insurance company as a cost savings measure

Risks	Risks to health	Contracting infection while abroad
	
	Risks of travel	Flying post-operatively
	
	Pre- and post-operative risks at home	Little legal recourse in certain jurisdictions

First-hand accounts	Positive and negative aspects of experiences	Reports of being satisfied with care received
	
	Sensationalized issues	Reports of needing to counter others' negative perceptions about destination countries
	
	Post-recovery life	Reports of improved health status

### Implications for Patients

From the sources reviewed for this scoping review it can be understood that patients are likely to hold significant responsibilities in the practice of medical tourism. For example, informational continuity of care is a quality indicator and is established through patient information being available over time and to multiple practitioners in different locations [105,106]. While patients often have roles to play in its establishment [107], medical tourists may hold particular responsibilities in this regard as they may literally be expected to transport hard copy records vast distances and ensure that they arrive safely to the correct people. Certainly, concerns are that these records may become damaged during transit or that the patient may choose not to share details of their procedure abroad with their regular doctor, thereby threatening informational continuity of care and the benefits it bestows.

Another responsibility is that it may be advisable for patients to take active measures to avoid encountering risks when traveling and also while abroad. While all patients run the risk of being exposed to any number of health threats when receiving surgery, such as surgical site infections and clotting complications [108,109], there can be additional risks that pertain to the 'travel' and 'receiving care abroad' dimensions of medical tourism that patients must take responsibility for minimizing or eliminating. Related to this, patients interested in medical tourism are also likely to hold responsibility for evaluating the trustworthiness and reliability of information sources (e.g., promotional materials, facilitators/brokers, friends and family). It has been said that international regulation of the medical tourism industry is lacking [110], and so patients are left to their own - possibly with the assistance of others - to rate and rank things such as the quality of facilities and procedure outcomes using available information. Clearly, it can be understood that how patients address these potential responsibilities is likely to directly shape their experiences of intentionally accessing medical care abroad via medical tourism.

This scoping review has revealed that the patient's experience of medical tourism might not end upon returning home, nor begin at the point of departure. In fact, it can extend far in advance of and beyond when care is received. The law reviews examined have focused extensively on the fact that there is typically little legal recourse for patients accessing surgeries and other procedures abroad in countries with weak malpractice laws. For patients who experience negative outcomes, their pursuit of compensation, whether financial or otherwise, may draw on over an extended period of time, quite possibly with little result, extending well beyond the post-operative recovery period.

It was noted in the findings section that word-of-mouth is likely to be important within the medical tourism industry: satisfied patients can spread information about facilities and destination countries to interested others. In this way, these medical tourists may become 'ambassadors' for destination countries and hospitals over time after returning home. In doing so they may effectively challenge perceptions of the health care delivered in some countries, thus working to overcome the kinds of 'straw hut' perceptions of care shared in the first-hand accounts subsection. At the same time, negative experiences of care abroad may serve to further entrench these perceptions.

### Knowledge Gaps

Although a significant number of sources were accepted into this scoping review, the vast majority did not present empirical research or other tested facts. Instead, this review has revealed that most of what is known about the patient's experience of medical tourism is, in fact, speculative, idea-based, or anecdotal in nature. The frequency with which some things were reported in the sources reviewed, such as cost savings as being a significant motivator for patient engagement and waiting lists pushing patients abroad, suggests that there is some consensus about specific aspects of the patient experience. However, often authors looked to media sources to verify such aspects given that empirical evidence is lacking. This is not surprising as newspaper and magazine articles were found to be some of the only sources that shared first-hand accounts from medical tourists, thus allowing for limited confirmation about ideas regarding motivations and other factors central to the patient's experience. Put another way, the area of medical tourism is ripe for research to be conducted by social and health scientists alike from a range of disciplinary and methodological perspectives. Research is needed not only to confirm or refute long-standing speculation regarding the patient's experience, but also to 'shed light' on areas that have been given almost no consideration to-date. For example, it seems that little attention has been paid to establishing the size and directions of the international flow of patients or the success rates of procedures performed at medical tourism hospitals. No doubt data access is a challenge, in that private hospitals treating international patients are likely reluctant to share such information.

A number of specific knowledge gaps are evident within the four thematic areas regarding the patient's experience of medical tourism identified in the scoping review. Research attention needs to be given to understanding how information sources consulted and evaluated by patients prior to departure. It would also be useful to better understand how patients understand the risks of accessing care abroad at this point in time. Retrospective accounts upon returning home could then shed light onto whether or not important factors were adequately considered in advance of ultimately deciding on accessing care abroad. Such research could offer useful insights into what patients need to know before going abroad and could ultimately assist with developing patient-focused decision-making aids. Significant knowledge gaps also exist regarding the push factors, pull factors, and motivations identified in the scoping review. Research needs to be conducted to confirm that those factors cited by the sources reviewed are indeed accurate, and also to determine whether or not others exist and also how they might differ between individuals and also patient groups (e.g., by procedure type, by home country, by destination country). In fact, any research that contributes to enhancing our understanding of how experiences of medical tourism differ between patients and the roles of factors such as socio-economic status, diagnosis, and overall health status in such differentiation is highly needed.

It was noted at the outset of this section that multiple individuals and groups are involved in shaping the patient's experience of medical tourism. As such, it is essential to turn research attention to these important stakeholders so as to better contextualize how patients experience this practice. This could include investigating doctors' and facilitators/brokers' roles in assisting patients during the decision-making process and also in sharing information on the risks. The practice of medical tourism holds significant implications for the airlines that transport patients and also travel agents who do bookings for patients not using the services of facilitators/brokers. There was almost no discussion of these important stakeholders in the sources reviewed in this scoping review. An understanding of the perspectives of these businesses and others who are linked to the industry (e.g., travel insurance, hotels) on the soundness of the practice of medical tourism and the ways in which it strengthens and/or threatens their operations is needed. This research would also be valuable because so very little consideration has been given to the tourism aspect of medical tourism. Because of this oversight, little is known about the importance of the tourism aspects of travel and decision-making, among other factors, for medical tourists. Tourism scholars, thus, have the ability to make important contributions to this area of health services knowledge.

## Limitations

A comprehensive approach was taken to scoping a variety of sources to synthesize what is known about the patient's experience of medical tourism. The inclusion of steps such as having two reviewers for every full source, developing a search strategy in consultation with a librarian, and searching for sources of all types from a comprehensive grouping of databases have added rigour to the scoping process and thus serve as strengths. However, two main limitations exist. The first is that only English-language sources were retrieved and reviewed. No doubt there is literature on medical tourism that has been produced in other languages. At the same time, there were very little non-English-language sources cited in the pieces reviewed, and so this suggests that the most important sources may be available in English. For example, industry and government reports produced in non-English-speaking countries were commonly available in English and so were included in the review process (e.g., [25]). The second main limitation is that the media sources reviewed were limited to Canada and a few major North American magazines and newspapers known to cover Canadian health and health care issues. Placing this restriction on the inclusion of media sources was necessary in order to keep the review manageable. As such, the media sources included are presented as a representation of the types of local, regional, and national coverage that exists of medical tourism within a particular country known as a departure point for medical tourists.

## Conclusions

Given the burgeoning, complex, and frequently controversial phenomena associated with medical tourism, this article has presented a much-needed scoping review to illustrate what is known (and not known) about this practice, especially in terms of patients' experiences. Addressing multiple types of literature, we hope this comprehensive knowledge synthesis will usefully guide research, government, and industry agendas alike. Seeking a focused understanding of medical tourism, full review of the 216 included sources (the overwhelming majority of which did not present empirical findings) identified four main themes that summarize what is known about the patient's experience. These themes are characterized by a focus on: (1) patients' *decision-making *in terms of push and pull factors, as well as the consultation of information; (2) *motivations *related to procedure, travel, and cost; (3) *risks *associated with patients' health, travel, and pre- and post-operative conditions in the home country; and (4) *first-hand accounts *of the positive and negative components of medical tourism, sensationalized issues, and post-recovery life. Using insights gleaned from the scoping review, we suggest that medical tourism is likely to have significant implications for patients, including that patients may very well hold a number of responsibilities should they choose to engage in intentionally going abroad for non-emergency care that is paid for out-of-pocket. Among these is potentially playing a role in ensuring that continuity of care is maintained as best as possible despite the disjuncture in their care trajectory across countries and providers.

Despite engagement with the issue of medical tourism in the published literature, it is clear that there is profound paucity of theoretical and empirical understandings of this practice that can ultimately help us to understand the patient's experience. With empirical evidence lacking, authors have frequently drawn on media sources to substantiate their claims. We believe that the time is ripe for social and health scientists from various disciplinary, theoretical, and methodological perspectives to go beyond enduring speculations about patients' experiences of medical tourism. As a starting point, we have identified a number of pressing research direction above. Finally, we hope the identified knowledge gaps and research challenges, along with the scoping review findings, illustrate the exciting possibilities for how scholars can make significant contributions to what is known about the patient's experience of medical tourism.

## Abbreviations

US: United States

## Competing interests

The authors declare that they have no competing interests.

## Authors' contributions

VAC led the identification of themes and writing of this manuscript. All authors contributed to the design of the scoping review, reviewing sources, and assisting with data management and interpretation. All authors provided feedback throughout the drafting of this article and have read and approved the final manuscript.

## Pre-publication history

The pre-publication history for this paper can be accessed here:

http://www.biomedcentral.com/1472-6963/10/266/prepub
